# The role of the liver in the migration of parasites of global significance

**DOI:** 10.1186/s13071-019-3791-2

**Published:** 2019-11-08

**Authors:** Gwendoline Deslyper, Derek G. Doherty, James C. Carolan, Celia V. Holland

**Affiliations:** 1 Department of Zoology, School of Natural Sciences, Trinity College Dublin, Dublin 2, Ireland; 2 School of Medicine, Trinity College Dublin, Dublin 2, Ireland; 3 Department of Biology, Maynooth University, Maynooth, Co. Kildare Ireland

**Keywords:** Liver, Migration, Immunotolerance, *Ascaris*, *Schistosoma*, *Plasmodium*

## Abstract

Many parasites migrate through different tissues during their life-cycle, possibly with the aim to enhance their fitness. This is true for species of three parasite genera of global importance, *Ascaris*, *Schistosoma* and *Plasmodium*, which cause significant global morbidity and mortality. Interestingly, these parasites all incorporate the liver in their life-cycle. The liver has a special immune status being able to preferentially induce tolerance over immunity. This function may be exploited by parasites to evade host immunity, with *Plasmodium* spp. in particular using this organ for its multiplication. However, hepatic larval attrition occurs in both ascariasis and schistosomiasis. A better understanding of the molecular mechanisms involved in hepatic infection could be useful in developing novel vaccines and therapies for these parasites.

## Background

The life-cycle of many parasites in their final host often involves migration from one tissue to another. The reasons for this are unclear; however, tissue migration has been linked to increased body size and maturation, which leads to improved parasite survival [1]. A recent study [2] suggested that tissue migration enables the parasite to avoid eliciting an immune response, which is often raised at mucosal surfaces. If evading the host’s immune response is in fact a key purposes of this migration, the potential role of the liver becomes clear. The liver is an immunotolerant organ and therefore an ideal place for parasites to hide from the immune system. We will use human parasites of three genera, *Ascaris*, *Schistosoma* and *Plasmodium*, to illustrate the essential role of the liver in the life-cycle of these parasites. We will demonstrate that the liver is a crucial step in their life-cycle, a point at which infection appears to go unnoticed, but a potential bottleneck where vaccination/treatment could be most effective.

## Immunotolerance in the liver

The special immune status of the liver was first identified in transplantation experiments in pigs, where allogeneic liver transplants were not rejected as was the case with other organs [3, 4]. In humans some tolerance is observed whereby transplanted livers recover spontaneously after a rejection reaction [4] and some liver allograft recipients can even be completely withdrawn from immunosuppression [5].

Hepatic immunotolerance occurs through a combination of unique anatomical and histological features of the liver. Most of the blood that enters the liver comes directly from the portal system, making it the first organ to be exposed to gut-derived molecules including harmless bacterial products and nutrients [6]. The smallest unit of the liver, the hexagonal lobule, consists of a small layer of hepatocytes around a central vein. The capillary bed of the liver, the sinusoids, does not form tight junctions, instead it forms fenestrations that are known as sieve plates [3]. The perisinusoidal space, also called the space of Disse, replaces the basement membrane to separate the liver sinusoidal endothelial cells (LSECs) from the hepatocytes [3].

The liver sinusoids are home to multiple populations of resident immune cells. These include myeloid leukocytes and liver parenchymal cells that express receptors that sense pathogens, and myeloid and lymphoid cells capable of phagocytosis and cytotoxicity [7]. Central to the tolerogenic nature of the liver, the sinusoids contain multiple populations of antigen-presenting cells (APC) which present antigenic peptides bound to major histocompatibility complex (MHC) molecules to T lymphocytes of the adaptive immune system [7–9]. Hepatic APCs are capable of activating T cells *in vitro*, inducing cytotoxicity and inflammatory cytokine secretion [7]. However, in the environment of the liver, hepatic APCs are more likely to inactivate T cells or induce their maturation into regulatory T (Treg) cells that suppress immune responses [3].

Antigen presentation to T cells is typically mediated by dendritic cells (DC) [10]. DCs express pathogen receptors that enable them to recognise components of microorganisms that are not found in mammalian systems. They also express costimulatory and/or coinhibitory receptors and release cytokines that determine the nature of T cell activation or T cell tolerance. Antigen presentation by hepatic DC generally results in T cell inactivation by anergy or exhaustion. These tolerogenic DC can also drive the differentiation of naïve T cells into Treg cells, which release immunosuppressive cytokines and suppress the activities of other immune cells in an antigen-specific manner [11–13].

Macrophages, known as Kupffer cells, are also abundant in the liver sinusoids. Similar to other macrophages, two subsets of Kupffer cells (KC), defined by their phagocytic and cytokine-producing properties, have been described [14]. ‘Inflammatory’ or M1 macrophages secrete high levels of the proinflammatory cytokine IL-12 and low levels of the regulatory cytokine IL-10, whereas ‘alternatively-activated’ or ‘repair’ M2 macrophages, produce high levels of IL-10, TGF-β and low levels of IL-12 [15]. Upon pathogen receptor ligation, KCs most frequently act as M2 macrophages [16] and antigen-presentation by these cells is frequently associated with expression of inhibitory ligands and cytokines and the induction of Treg cells [17, 18].

LSECs also express MHC and costimulatory molecules and are capable of presenting antigen to CD8^+^ T cells leading to tolerance [19–21] and to CD4^+^ T cells leading to their differentiation into Treg cells [22]. Hepatic stellate cells (HSC), also called Ito cells, can also present antigen to T cells [23], but again, antigen presentation by this APC preferentially promotes T cell tolerance [24, 25]. HSC can also promote the differentiation of monocytes into myeloid-derived suppressor cells (MDSC), which have potent T cell inhibitory activities [26]. Hepatocytes also express pathogen receptors, MHC and costimulatory molecules, although it is not clear these cells can present antigens to T cells leading to their activation [27, 28].

## Three parasite genera of global importance: *Ascaris*, *Schistosoma* and *Plasmodium*

**Fig. 1:**
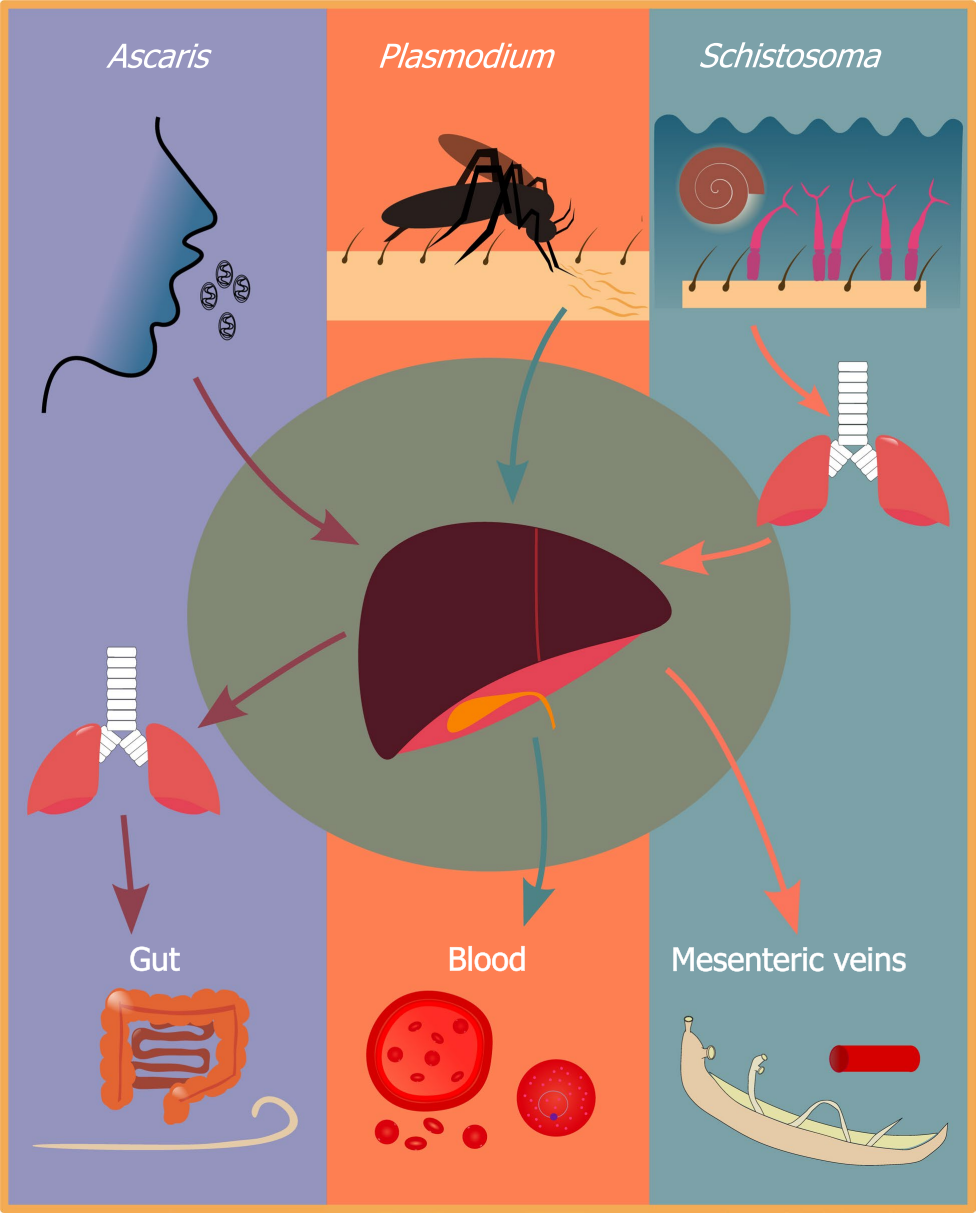
The migratory path of *Ascaris*, *Plasmodium* and *Schistosoma. Ascaris* eggs are ingested orally and after hatching, larvae penetrate the gut wall. The larvae subsequently move to the liver and the lungs from where they are coughed up and swallowed thereafter establishing as adult worms in the gut. *Plasmodium* sporozoites are injected into the skin; from there they migrate to the liver where they multiply into merozoites. Merozoites leave the liver and infect erythrocytes where they eventually mature into gametocytes to continue the life-cycle. Schistosome cercariae are released into water from infected snails. Cercariae penetrate the skin and then migrate to the lungs and then the liver. Ultimately, these parasites establish themselves as adult worms in the mesenteric veins

Species of the three parasite genera discussed in the present review all use different ways to enter their final hosts, oral ingestion of eggs (*Ascaris* spp. [29]), skin penetration by free-swimming cercariae (*Schistosoma* spp. [30]), and injection into the blood stream *via* mosquito bites (*Plasmodium* spp. [31]) (see Fig. 1). Despite entering different tissues, the parasites migrate to the liver rather quickly.

After egg-hatching in the gut, *Ascaris* spp. larvae are transported to the liver *via* the portal vein. Subsequently the larvae migrate to the lungs, where they are coughed up and swallowed thereby re-entering the gut [32]. The complexity of this life-cycle, in addition to the fact that both the start and end organ of the life-cycle are one and the same, the gut, indicates the importance of tissue migration, potentially related to increased fitness of the parasite.

*Schistosoma* spp. are slightly different, as the parasites migrate to the a different organ, the lungs first, before entering the liver and ultimately reaching the mesenteric vessels [30]. This is therefore the opposite path that *Ascaris* spp. larvae take. Although *Schistosoma* spp. reach the lungs first and the liver second, we will show that the role of the liver in the migratory path is still very important in the larval development. In particular, in non-immune animals, the liver appears to play an important role in parasite attrition.

*Plasmodium* sporozoites are injected into the skin by an infected mosquito and migrate to the liver *via* the bloodstream [33] where the parasites mature into merozoites and multiply. Subsequently the merozoites are released in great numbers into the bloodstream and infect erythrocytes where they mature into trophozoites, schizonts which release merozoites during asexual multiplication [33]. Ultimately trophozoites will mature into male and female gametocytes that can be picked up by a mosquito bite. The liver is therefore used by the parasite as a ‘safe haven’ for the parasites to evade the hosts’ immune system. It is also the place where hypnozoites from *P. vivax* can remain unnoticed for years before restarting their life-cycle and causing malaria pathology.

## *Ascaris*: self-cure and differential burden

*Ascaris lumbricoides* is a soil-transmitted helminth which infects 800 million people worldwide [34]. The eggs have a thick shell, making them highly resilient to various environmental factors such as temperature and desiccation [35]. Infections usually peak in children between 5–15 years-old, who can experience symptoms ranging from growth retardation to diminished cognitive development [35, 36].

Most infected individuals carry light worm burdens but a relatively small proportion harbour heavy infections, a distribution described as aggregated [37]. Predisposition to infection has also been observed in ascariasis, a phenomenon whereby individuals are prone to a particular intensity of infection and regain similar worm burdens after treatment [38]. Although the exact mechanism of predisposition is unknown, it has been found to involve various factors, such as host genetics and adaptive immunity [39].

While the liver stage appears to be clinically silent, the presence of macroscopical white spots, areas of inflammation formed around the larvae due to injury during migration, in the liver of both humans infected with *A. lumbricoides* [40] and pigs infected with the porcine ascarid *A. suum* [41] is a clear indication that an immune response is generated. When treating experimentally infected pigs with anthelmintics during the liver stage (day 2, 3 and 4 post-infection, p.i.) of *A. suum* infection, an increase of 22% in their feed conversion rates, was found when compared to treating the pigs at the lung stage of infection (day 6, 7 or 8 p.i. [42]). As for humans, a prospective study of 510 Indian patients with liver abscesses during a 10-year period identified *A. lumbricoides* as the causative agent in 14.5% of the cases [40]. Similarly, a study in South African children demonstrated that *A. lumbricoides* was the causative agent of liver abscesses in 2% of the cases [43].

## Self-cure in pigs

Self-cure is a phenomenon observed in some pigs, where pigs receiving an oral trickle infection with *A. suum* eggs do not develop intestinal worms. The potential role of the liver in this phenomenon has received considerable attention.

In order to assess the role of the liver, an experiment was performed which bypassed the liver by infecting pigs with L3 stage (the lung stage) larvae through intravenous injection [44]. At day 21 p.i., a time at which self-cure usually has occurred, pigs did not show the typically observed decreased larval burden. This led the authors to believe that the liver played a crucial role in self-cure. However, the lack of a control group of orally infected pigs makes it hard to confirm this hypothesis. Another study approached the question of the role of the liver in self-cure by orally infecting pigs with lung-stage larvae [45]. The authors found first that self-cure still occurred, and secondly, that it happened at the same rate as their controls, i.e. pigs orally infected with embryonated eggs. A weep and sweep response has been attributed to this observation, whereby increased mucus secretion and gut movement eliminates the larvae before they can penetrate the gut wall [46]. Additionally, intestinal eosinophils and T cells were found to potentially play an important role. The mechanisms behind self-cure appear to be diverse and are not yet fully understood.

## A mouse model for hepatic resistance

In order to study the liver stage of ascariasis, an appropriate animal model is necessary. Building on earlier work [47], a mouse model has been developed specifically to study the predisposition phenomenon by using two inbred mouse strains which mimic the extremes of predisposition to light and heavy infection [36, 48, 49]. CBA/Ca and C57BL/6J were found to have a substantial and consistent difference in lung larval burden, with the former as the relatively resistant strain and the latter as the relatively susceptible strain.

This mouse model was subsequently used to investigate when, during migration, this difference in larval burden occurs [50]. Mice of both strains were infected with the same number of *A. suum* eggs. At regular intervals from 6 hours p.i. until 8 days p.i., mice were sacrificed and their organs (gut, liver, lungs) removed. The larvae were retrieved from those organs, using the modified Baermann method. The larvae were subsequently enumerated and counted. This allowed for detailed tracking of larval migration throughout the various organs. Using this mouse model, similar larval burdens were found in the liver of both strains [50]; however, once the larvae reached the lungs a significant difference in larval burden was observed, with the relatively resistant strain having a lower burden than the relatively susceptible strain. Another study found similar results, with no difference in total larval burden in the liver between the two strains [49]. The first larvae appear in the liver at 6 hours p.i. with a peak appearing between days 3–6, at this stage the larval burden is similar in both strains [50]. The larvae subsequently migrate to the lungs, with the first larvae appearing as early as day 1 p.i.; however, the majority of the larvae arrive around day 6 p.i. At this point it becomes clear that one strain is relatively susceptible (C57BL/6J) and another relatively resistant (CBA/Ca), as there is a statistically significant difference in lung larval burdens between the two strains. Interestingly, the authors pointed out that there is a steady increase in mean larval burden in the liver between days 2 and 5 p.i., long after the larvae should have penetrated the gut wall and made their way to the liver. The authors speculated that “larvae were arriving from other locations and perhaps had become temporarily lost or trapped in other host tissues”, indicating a strong instinct for the larvae to make their way to the liver and highlighting the importance of this organ during migration.

The question then remains, which organ contributes most to this difference in infection rate, is it the departing organ, the liver, or the organ where the larvae arrive, the lungs. In order to explore this question in more detail, an investigation of the presence of various leukocytes in the bronchoalveolar fluids (BAL) in both strains of mice, infected with *A. suum* [50]. This study showed that an increase in leukocytes occurred at days 8–9 p.i., so only after the difference in larval burden had already occurred. Additionally, it was found that the increase in leukocytes in BAL was higher in the susceptible strain than in the resistant strain, indicating that this increase did not contribute to the difference in larval burden between the two mouse strains but reflected the observed larval burden. This was confirmed in the same study by examining the lung tissue, in which was observed a greater inflammatory response in the susceptible strain. Taken together, these data indicate the importance of the liver stage and that it plays a key role in the difference in infection rate between these two mouse strains.

Shotgun mass spectrometry was used to explore the liver proteome in the above mentioned mouse model at day 4 p.i. [51]. We observed a difference in abundance of mitochondrial proteins involved in oxidative phosphorylation. The relatively resistant strain (CBA/Ca) had, both intrinsically and under infection, higher levels of this protein group than the relatively susceptible strain (C57BL/6J). This led us to believe that a potential intrinsic difference in reactive oxygen species (ROS) in the liver gives the relatively resistant strain an advantage in contending with the parasite.

A subsequent study investigated the difference in liver proteome at day 7 p.i. This study confirmed the earlier findings of day 4 p.i., demonstrating that the relatively resistant strain had a higher abundance of proteins involved in oxidative phosphorylation. However, the later day experiment revealed an important difference in the immune response to *A. suum* in the liver, with the relatively susceptible strain showing a higher abundance of proteins involved in complement inactivation compared to the relatively resistant strain which had a higher abundance of proteins involved in complement activation [52]. These two studies demonstrate the importance of delineating host responses to helminth infection at different time points post-infection.

During a reinfection experiment [53], BALB/c mice were infected with *A. suum*, the authors found no difference in the liver larval burden when comparing mice that were infected once or reinfected. However, a lower larval burden was observed in the lungs in the reinfected mice, and more importantly, lesions caused by hepatocyte necrosis and infiltration of eosinophils and neutrophils were more pronounced in the reinfected group. These results indicate that the observed, more pronounced hepatic immune response in the reinfected group results in a decrease in lung larval burden.

To conclude, the presence of white spots indicates the presence of a strong immunological response in the liver. The liver stage becomes particularly important when investigating predisposition in pigs, mice and humans. Novel therapies could therefore focus on the liver in order to stop larval migration in its tracks, prevent increased damage due to larval migration and ultimately inhibit the development of adult worms which have significant impact on the nutritional and developmental status of the host [51, 54].

## The liver as a site for attrition in *Schistosoma* infection

Species of *Schistosoma* infect over 250 million people worldwide [30]. Reinfections are common in endemic regions, with children often having suffered their first infection by the age of two, followed by a steady increase in worm burden with every new infection [55]. After these early infections, the worm burden decreases with increased exposure to the parasite that is thought to increase immunity, combined with the death of older worms [30].

*Schistosoma* spp. interact with the liver during two distinct parts of their life-cycle. First, for all human *Schistosoma* species, the immature worms pass through the liver’s vasculature after lung migration. Secondly, after the migratory path has been completed and the adult worms are located in the mesenteric vessels, some excreted eggs however do not end up in the faeces but instead travel to the liver *via* the hepatic vessel, this is true for all human *Schistosoma* species except *S. haematobium*. *Schistosoma haematobium* is the only species that does not cause liver pathology, as the adult worms are located in the *venus plexus* of the bladder [56]. In order to break down the eggs, granulomas are formed around them, causing hepatic fibrosis, which is the main source of mortality and morbidity in chronic schistosomiasis [57]. Liver-associated disease in schistosomiasis is more common in *S. japonicum* and *S. mansoni* infections [58]. The liver is also profoundly impacted due to the longevity of the adult worms with an average life span of 3–10 years [59]. In essence, the most damaging symptoms associated with *Schistosoma* spp. infection, hepatic granuloma formation does not occur until after larval migration is completed and can be considered an unwanted side effect.

## The liver as a site for maturation, pairing and sexual development

The liver stage is a crucial phase in the life-cycle of schistosomes, it is here that they can increase their biomass and develop into mature life stages [60, 61]. These important life-cycle changes do not occur at any other stage, in fact, schistosomulae of *S. mansoni* which were trapped in the pulmonary vasculature were not able to reach maturity [60, 62]. Parasites that reached the liver, however, showed exponential growth, thus demonstrating that the liver’s vasculature alone can provide the parasites with an adequate environment to reach maturity. Additionally, it was found that culturing *S. mansoni* schistosomulae in the presence of human portal serum showed a significant increase in cell proliferation when compared to schistosomula cultured in the presence of human peripheral serum [63, 64], again confirming that the liver vasculature provides the optimal environment for these parasites. However, so far it is not clear which components contribute, or might be essential for these processes to occur.

Additionally, it is at the liver stage that male and female parasites pair, which allows the females to reach sexual maturity; females cannot reach sexual maturity without the presence of males [65]. The paired schistosomes subsequently migrate against the blood flow to the mesenteric veins [66]. The fact that these parasites need to manoeuvre against the blood flow, a process that female parasites cannot do by themselves [66] highlights again the importance of the liver stage for this parasite.

## Attrition: when, where and how?

The site of attrition has been a topic of contention for decades, it was studied intensively in the 1980s thanks to the development of the autoradiographic tracking [67] which greatly improved the sensitivity of these experiments. Using this technique, it was found that between 86% and 90% of the skin-penetrant cercariae in naïve mice (i.e. mice that received a single round of infection) had migrated to the lungs, indicating that the skin was not the site of attrition [68, 69]. Further investigation of the migration of *S. mansoni*, using autoradiographic tracking in C57BL/6 mice showed a peak in the lungs at day 8 and in the systemic organs at day 12 [70]. All this indicates attrition occurring between the pulmonary and hepatic life stages of the parasite [68]. This raises the question as to why these parasites move to the liver next - perhaps the liver is a safe haven - a place where the parasites are no longer under attack and can reach sexual maturity safely hidden from the immune system.

As for immunized animals, which were immunized through either gamma-irradiated cercariae or establishing a chronic infection, the attrition is largely similar to that of naïve animals, except that everything appears to go more slowly, i.e. migration from the skin to the lungs takes longer and peak burden in the lungs was at a later time point. The big difference with naïve animals is the lower number of parasites reaching the systemic and splanchnic organs [70–73].

The location of attrition varies between *Schistosoma* species. For *S. mansoni* and *S. haematobium* attrition mainly occurs in the lungs [74, 75]. However, five times more *S. mansoni* larvae reached the liver than is the case for *S. haematobium*. This observed greater larval death in *S. haematobium* infection can be partially attributed to the fact that mice are non-permissive hosts for this parasite [76]. Attrition in *S. japonicum* was found to occur in both the lungs and the liver [77].

Regardless of whether the lungs or the liver are the main site of larval removal, it is still crucial to investigate the role of the liver in the life-cycle, which could also be a potential target for vaccination. Early removal of the parasite, before it reaches sexual maturity and therefore egg-laying, would reduce granuloma formation.

## Multiplication of *Plasmodium* in the liver

The protist parasites *Plasmodium* spp. are the causative agents of malaria in humans, resulting in an estimated 429,000 deaths in 2015 [78]. There are five species that infect humans: *P. falciparum*, *P. ovale*, *P. vivax*, *P. malariae* and *P. knowlesi*. Of these, *P. falciparum* infection is the most common and pathogenic, causing 99% of malaria associated deaths [78].

Malaria-associated pathology is often divided into uncomplicated and severe *P. falciparum* malaria. Uncomplicated malaria results in general malaise [31]. Severe falciparum malaria is caused by *P. falciparum* sequestering in the small and medium-sized blood vessels of different organs particularly the brain [31]. Pathology in humans is therefore mainly associated with the blood stages of the parasite and complications thereof such as cerebral malaria [31]. The lack of symptoms and the brief duration of the liver stage, make it a particularly difficult stage to study. However, a recent study using malaria-naïve human volunteers who were infected with *P. falciparum* through infective bites observed an increase in total leukocyte, lymphocyte and monocyte count during the liver stage followed by a decrease in the aforementioned counts when the blood stage is initiated [79].

The liver is the only organ necessary for *Plasmodium* spp. maturation. At this stage a low number of sporozoites proliferate into a large numbers of merozoites, ready to invade erythrocytes and start the process of the well-known cyclic fever bouts. Due to this bottleneck, the liver stage forms an ideal target for vaccination purposes. However, before a vaccine can be developed, an in-depth understanding of this stage is necessary. The National Institute of Allergy and Infectious Diseases recently identified ‘greater understanding of parasite liver-stage biology and development’ as a key challenge in malaria vaccine development [80].

## The path to the liver

After being deposited in the skin from a mosquito bite, sporozoites migrate to the liver *via* the blood. To enter the liver, the sporozoites must cross the fenestrated endothelial layer; however, the fenestrations are too small for the sporozoites to pass through, thus passage must go through the sinusoidal cells [81]. Circumsporozoite protein (CSP) and thrombospondin-related anonymous protein (TRAP), expressed by sporozoites, binds human heparin sulphate proteoglycans (HSPGs), the signal for sporozoites to leave the blood stream [82, 83]. Whether the sporozoites invade the liver through KC invasion or not is still under debate, with some studies suggesting a necessary step through KCs [84, 85], whereas others indicating that hepatocytes can be invaded directly [86]. Some studies suggest that sporozoites need to travel through KCs, whereas others indicating that hepatocytes can be invaded directly. Sporozoites have been shown to directly infect hepatocytes *in vitro* and develop into merozoites; however, due to the cellular structure of the liver it has been suggested that *in vivo* the sporozoites must pass through KCs [84]. Frevert et al. [85] described the migration of the sporozoites with an abrupt speed change, at which point they glide along LSECs, followed by a long pause for the sporozoites to enter the KCs, which the authors attribute to formation of nonfusogenic vacuole formation and a relatively slow passage through the KCs. CSP from sporozoites has been shown to elevate cAMP levels in KCs, thereby inhibiting the cells from producing a respiratory burst and thus protecting the sporozoites [87]. Additionally, sporozoite microneme protein essential for cell traversal 2 (SPECT2) deficient sporozoites were not able to infect the liver *in vivo* [88]. These data suggest the need of KCs in hepatocyte infection.

However, a recent study provided evidence for the alternate hypothesis [86]. The authors, building on earlier work [89], identified that 17% of sporozoite cell traversals exclusively involve endothelial cells [86]. They also identified that 15% of crossing events were independent of cell traversal and KCs, which could be the sporozoite moving between two endothelial cells or between an endothelial cell and a KCs. Furthermore, the authors found that sporozoites KCs traversal is associated with cell death of those KCs.

Regardless of this, sporozoites will eventually migrate through several hepatocytes, for yet unknown reasons, using a transient vacuole to ensure passage [85]. Three theories exist as to why the *Plasmodium* parasites travel through several hepatocytes before forming the PV and ultimately differentiating into merozoites. The first theory argues several rounds of migration through hepatocytes might be a protective mechanism by the parasite to ensure that formation of the PV and subsequent merozoites formation only occurs in the liver and not in the other tissues (skin, etc.) [89]. A second theory builds on the observation that hepatocyte growth factor is released when the parasite migrates through hepatocytes this induces neighbouring hepatocytes to be more susceptible to infection [90]. Thirdly, it has been suggested that upon detection of high sulphated HSPGs, the sporozoites turn off their traversal machinery and activate the invasion machinery; however, this has been shown to take between 30–60 min in *Plasmodium yoelii*, which would explain the invasion of multiple hepatocytes [91, 92]. When reaching their final hepatocyte, a parasitophorous vacuole (PV) is formed and the sporozoites can differentiate into merozoites [85].

The merozoites will then need to pass the space of Disse, at which stage they are vulnerable to KC phagocytosis [93]. To avoid this, the infected hepatocytes release merozoite-filled vesicles, which are derived from the plasma membrane of hepatocytes, into the liver sinusoids [93, 94]. The phosphatidylserine switch is prevented in these merozoites-filled vesicles so as not to alert the KC and other immune cells, granting an escape route for the parasite [93].

## Hypnozoites

Hypnozoites have only been observed in *P. vivax* [95], although a handful of cases of relapsing *P. ovale* have been seen as well, but without confirmation of hypnozoites [96]. This life stage arguably exploits the liver’s special immune status to the fullest. By going into a dormant state in the liver, for weeks or months, these parasites can hibernate and resume infection [97]. No study so far have been able to identify the triggers for hypnozoites reactivation, nor its specific relationship to the liver.

The importance of these hypnozoites, which can remain dormant for 6–9 months, cannot be overstated if the ultimate goal is malaria elimination [98, 99]. Up to 80% of all blood stage *P. vivax* infections are attributed to relapses; however, not all relapses can be attributed to hypnozoites and other mechanisms are thought to be at play [100–102]. Investigating these hypnozoites more in depth could give a better insight in to why *Plasmodium* spp. incorporate the liver in the life-cycle and how they exploit hepatic immune tolerance.

## Hepatic immunity

Naturally acquired immunity is probably not achieved at the liver stage, rather it is more likely an antibody-mediated response to the blood stage [33]. However, the liver stage offers a great opportunity to activate the immune system and eliminate the relatively few sporozoites before they multiply greatly in numbers and spread all over the body via the blood.

Innate recognition of *Plasmodium* RNA by melanoma differentiation-associated gene 5 protein (MDA5) and mitochondrial antiviral signalling (MAVS) pathway in infected hepatocytes induces activation of the transcription factors interferon-regulatory factors-3 (Irf3) and IRF7 [103]. Additionally, a recent *in vivo* study showed that protection against clinical malaria in children is associated with C1q-fixing antibodies against CSP in *P. falciparum* sporozoites [104]. These antibodies inhibited hepatocyte cell traversal and ultimately induced sporozoite death.

CD8^+^ T cells play an important role in immunity to malaria, with CD8^+^ T-cell depleted mice being unable to develop immunity [105]. The efficacy of parasite inhibition is therefore dependent on the availability of effector CD8^+^ T cells [106]. Some CD8^+^ T cells get primed by dendritic cells in the skin draining lymph nodes before moving to the liver where they eliminate antigen presenting hepatocytes [107]. No evidence exists that hepatocytes can successfully present antigen and activate naive CD8^+^ T cells, however, hepatocytes can present a CSP epitope of *P. berghei* to primed CD8^+^ T cells [106].

To ensure full inhibition of blood stage malaria development 100% immunity is required. To achieve this, the very low number of hepatocytes that are actually infected has to be fully eliminated in the relatively short hepatic period, meaning that there is a need for a very large threshold of memory T cells to ensure immunity after epitope-specific immunization [106].

In short, the immune response in the liver is not fully elucidated yet and knowledge appears to be lacking. *Plasmodium* spp. likely exploit the immunotolerance of the liver to increase the relatively small number of sporozoites to a much larger number of merozoites. The liver stage therefore forms an ideal target for vaccine development. If successful, a vaccine would be able to stop the infection before any symptoms occur and would eliminate the spread of malaria, thus also contributing to its elimination.

While the liver offers a immunotolerogenic environment for the maturation of species of *Ascaris*, *Schistosoma* and *Plasmodium,* it should be noted that the liver has many other attributes that make it an attractive residence for these parasites. The liver sinusoids comprise a network of capillaries containing nutrient-rich blood from the intestine. The low blood pressure in the sinuses may offer an environment that supports growth, maturation and/or multiplication. Furthermore, the liver has a unique ability to regenerate and remodel itself, an attribute which could be exploited by the parasites to limit the deleterious effects of infection and inflammation and preserve their host tissue. The propensity of the liver to induce tolerance of foreign antigens, rather than immunity, is another attribute that may attract parasites, as well as other microorganisms, to this organ.

## Conclusions

Immunologically the liver is a special organ where immune activation is reduced. This forms the ideal environment for species of *Ascaris*, *Schistosoma* and *Plasmodium* to mature to their next life stages, and multiply as is the case for *Plasmodium* spp. only. When migrating to the liver, the parasites are able evade the immune system. However, this does not always go according to plan. In the case of both *Schistosoma* spp. and *Ascaris* spp., the liver has been identified as a site of larval attrition. Understanding the molecular mechanisms behind this attrition could lead to the development of novel therapies. As for *Plasmodium* spp., the liver is a true bottleneck. It is at this life stage that the parasites multiply rapidly, before being released in the blood and spreading all over the host. The liver stage is therefore the ideal vaccine/drug target, as the parasite is still in relatively low numbers and concentrated in one organ. In short, the liver stage is understudied and more research is necessary to fully understand the molecular mechanisms and immune responses activated during parasitic invasion.

## Data Availability

Not applicable.

## Abbreviations

LSECs: liver sinusoidal endothelial cells
APC: antigen-presenting cells
BAL: bronchoalveolar fluids
Treg: regulatory T cells
DC: dendritic cells
KC: Kupffer cells
TLR: Toll-Like receptor
MHC: major histocompatibility complex
HSC: hepatic stellate cells
MDSC: myeloid-derived suppressor cells
ROS: reactive oxygen species
CSP: circumsporozoite protein
TRAP: thrombospondin-related anonymous protein
HSPGs: human heparin sulphate proteoglycans
SPECT2: sporozoite microneme protein essential for cell traversal 2
PV: parasitophorous vacuole
MDA5: melanoma differentiation-associated gene 5 protein
MAVS: mitochondrial antiviral signalling
Irf: interferon-regulatory factors
